# Efficacy of concentrated growth factor (CGF) in the surgical treatment of oral diseases: a systematic review and meta-analysis

**DOI:** 10.1186/s12903-023-03357-5

**Published:** 2023-10-04

**Authors:** Liang Chen, Jing Cheng, Yu Cai, Jingran Zhang, Xiaohui Yin, Qingxian Luan

**Affiliations:** 1 Department of Periodontology, National Center for Stomatology & National Clinical Research Center for Oral Diseases & National Engineering Laboratory for Digital and Material Technology of Stomatology, Peking University School and Hospital of Stomatology, No. 22, Zhongguancun South Avenue, Haidian District, Beijing, 100081 PR China; 2 Stomatological Hospital of Xiamen Medical College, Xiamen Medical College, Xiamen, PR China; 3 Xiamen Key Laboratory of Stomatological Disease Diagnosis and Treatment, Xiamen, PR China; 4 First Clinical Division, Center for Stomatology & National Clinical Research Center for Oral Diseases & National Engineering Laboratory for Digital and Material Technology of Stomatology, Peking University School and Hospital of Stomatology & National, No. 22, Zhongguancun South Avenue, Haidian District, Beijing, 100081 PR China

**Keywords:** Concentrated growth factor, Dental diseases, Dental implant, Periodontal diseases, Alveolar ridge preservation, Tooth extraction

## Abstract

**Background:**

Concentrated growth factor (CGF), a new autologous platelet concentrate, has been widely investigated to the adjunctive treatment of oral diseases. This study aims to evaluate the efficacy of CGF in the surgical treatment of oral diseases.

**Methods:**

MEDLINE, Web of Science, Scopus, Cochrane, and EMBASE databases were searched up to July 2023. Only randomized clinical trials were included. The methodologic quality was evaluated by the Cochrane Risk of Bias Tool. RevMan 5.4 software was used for data analysis.

**Results:**

In the treatment of periodontal intrabony defects, bone graft combined with CGF was significantly superior to bone graft (P < 0.01), with mean intrabony defect depth reduction of 1.41 mm and mean clinical attachment level gain of 0.55 mm. In the regenerative surgery of furcation defects, the effect of CGF group was significantly better than control group (P < 0.0001), with mean probing depth reduction of 0.99 mm, vertical bone gain of 0.25 mm, and horizontal bone gain of 0.34 mm. CGF combined with coronally advanced flap (CAF) was more effective than CAF alone (mean keratinized tissue width increase of 0.41 mm, mean gingival thickness increase of 0.26 mm, P < 0.00001), but less effective than connective tissue graft (CTG) combined with CAF (mean root coverage difference of -15.1%, mean gingival thickness difference of -0.5 mm, P < 0.0001). In the alveolar ridge preservation, additional use of CGF reduced horizontal bone resorption by 1.41 mm and buccal vertical bone resorption by 1.01 mm compared to control group (P < 0.0001). The VAS score of CGF group was significantly lower than that of the control group at the 1st and 7th day after oral surgery (P < 0.0001).

**Conclusions:**

CGF can exert a positive adjunctive effect for the regenerative surgery of periodontal intrabony defects, furcation defects, and alveolar ridge preservation procedure. CGF combined with CAF has a better therapeutic effect on gingival recession compared to CAF alone, although it is not as effective as CTG combined with CAF. CGF could promote postoperative healing and pain relief in oral surgery within a week. There is currently not enough evidence to support the clinical benefits of CGF in other oral surgeries.

**Supplementary Information:**

The online version contains supplementary material available at 10.1186/s12903-023-03357-5.

## Background

Oral diseases are a global public health problem, which include a range of clinical conditions that affect the teeth and mouth [1], including dental diseases, periodontal diseases, oral mucosal diseases, salivary gland diseases, jaw diseases, temporomandibular joint disorders, congenital oral anomalies, oral infections and oral cancers. Oral soft and hard tissue loss resulting from periodontal diseases, tumors, implant-related diseases, alveolar cleft, and alveolar bone atrophy after tooth loss, seriously affects mastication, occlusion, aesthetics, and mental health of patients. Tissue regeneration in the oral and maxillofacial region involves a variety of complex tissues, such as alveolar bone, dentin, cementum, gingiva, and oral mucosa. Recently, a plethora of different surgical techniques and biomedical materials, usually including guided tissue / bone regeneration (GTR / GBR), allografts, xenografts, synthetic graft materials, growth factors, enamel matrix proteins or various combinations thereof, have been employed to regenerate oral and maxillofacial tissues [2, 3].

Autologous platelet concentrates, which release considerable quantities of growth factors that can stimulate and promote bone repair and tissue healing, have been extensively investigated over the last several decades for oral and craniofacial regeneration [4, 5]. Platelet-rich plasma (PRP) is the first generation of platelet gels for oral and maxillofacial surgery [6], mainly prepared by a two-step centrifugation procedure and the addition of bovine thrombin and calcium to trigger platelet activation and fibrin polymerization [7, 8]. Platelet-rich fibrin (PRF) is the second generation of platelet concentrates, developed by Choukroun et al. [9], and is prepared using a simplified protocol than that of PRP and does not require the addition of anticoagulants, thrombin and calcium chloride [7]. Kobayashi et al. [10] have demonstrated that PRF is more potent in angiogenesis than PRP. Besides, the benefits of PRF in periodontal tissue regeneration have been reported in several systematic reviews and meta-analyses [11–14].

Concentrated growth factor (CGF) is the latest generation of autologous platelet concentrate, developed by Sacco in 2006 [15, 16], and is prepared by centrifuging blood samples with a special centrifuge device (Medifuge, Silfradent srl, Italy) [15]. Different centrifugation speeds of CGF permit the isolation of a fibrin matrix that is much larger, denser, and richer in growth factors fibrin matrix than PRF [15]. Studies reported that CGF and PRF have similar mechanical properties, degradability, and major growth factors contents, both of which are better than PRP [17, 18]. Moreover, PRF and CGF have the ability to stimulate a continual and steady release of total growth factors over a 14-day period and showed a similar effectiveness in periodontal bone regeneration [19]. Nevertheless, some findings showed the advantages of CGF compared to other platelet concentrates. According to Lee et al. [20], compared with PRF, tensile strength and growth factor contents of CGF were significantly higher. Li et al. reported that CGF showed more effective bone induction and tissue regeneration ability in the long term than PRP and PRF [21]. Hu et al. [22] demonstrated that CGF treatment improved the survival and quality of fat grafts, significantly better than PRP and PRF.

CGF can promote cell proliferation, migration, and differentiation [23, 24], as well as angiogenesis [25] and osteogenesis [26], all of which show great potential in tissue regeneration. CGF has been investigated to be effective in the treatment of bone defects [19, 27], implantology [28], gingival recession [29] and temporomandibular disorders [30]. However, due to insufficient randomized controlled trials (RCTs) for meta-analysis, only a few reviews [31–35] have reported the effect of CGF on oral and maxillofacial tissue regeneration. Subsequently, a growing number of RCTs have been published, allowing for a meta-analysis to be conducted on the efficacy of CGF in oral surgery.

The main objective of this systematic review and meta-analysis is to evaluate the additional benefits that CGF may provide for the treatment of oral diseases. Furthermore, we aim to evaluate the effect of CGF on postoperative healing and pain relief in oral surgery. By conducting this comprehensive analysis, we strive to enhance our understanding of the potential advantages of CGF in oral diseases.

## Methods

### Protocol and registration

The protocol of the present systematic review and meta-analysis was registered on the PROSPERO database (CRD42020206056). This study was conducted based on the guidelines of the Cochrane Handbook for Systematic Reviews of Interventions [36], and it is reported in accordance with the Preferred Reporting Project Guidelines for Systematic Review and Meta-analysis (PRISMA) statement [37].

### Eligibility criteria

The inclusion criteria were set following PICOS question:

Participants (P): Systemically healthy adults with surgically treatable oral diseases, including periodontal diseases, implant-related problems, periradicular lesions, post-extraction, jawbone defect and other oral diseases requiring surgical treatment.

Intervention (I): Oral surgery with the use of CGF as sole biomaterial or in combination to other biomaterials.

Comparison (C): Oral surgery without the use of CGF and other autologous platelet concentrates.

Outcomes (O): Alveolar bone and/or soft tissue wound healing, including radiographic and clinical parameters and patient-reported outcome measures. For the treatment of periodontal intrabony defects, primary outcomes were intrabony defect (IBD) depth reduction and clinical attachment level (CAL) gain, and secondary outcome was probing depth (PD) reduction. For the treatment of gingival recession, primary outcome was mean root coverage (MRC), and secondary outcomes were keratinized tissue width (KTW) increase and gingival thickness (GT) increase. For the treatment of furcation defects, primary outcomes were horizontal and vertical radiograph bone gain, and secondary outcome was PD reduction. For the alveolar ridge preservation, primary outcomes were ridge width changes and vertical bone resorption. For the effects on postoperative healing and pain relief, Landry healing index (Landry HI) and VAS score were regarded as the outcomes.

Study (S): Randomized controlled trials (RCTs), and only the study with the longest follow-up was included when study series used the same population.

The exclusion criteria were as follows: (1) patients with systematic diseases affecting oral diseases; (2) animal and in vitro research, reviews, non-randomized controlled trials, cohort and cross-sectional studies, case series and case reports; (3) insufficient/unclear data; (4) studies not evaluating the additional effect of CGF in the oral surgery; (5) no outcome of interests.

### Information sources and search strategy

An electronic search without limitation in language was performed in five electronic databases: National Library of Medicine (MEDLINE-PubMed), EMBASE, and Cochrane Library, Web of Science, and Scopus. The search terms and strategy are shown in Appendix 1. The last search was conducted on 18th July 2023. In addition, the grey literature was searched in the OpenGrey (http://www.opengrey.eu) and Grey Literature Report (http://www.greylit.org) by using the term “concentrated growth factor”. Furthermore, all reference lists of included papers and related reviews were searched to find possible additional studies.

### Study selection and data collection process

Two reviewers (L.C. and J.C.) independently screened the titles and abstracts of articles obtained from the initial search. Subsequently, both reviewers examined the full texts of all eligible articles. Publications that did not meet the inclusion criteria were excluded upon reviewer’s agreement. Any disagreement regarding inclusion or exclusion of the retrieved papers was resolved by open discussion between the two reviewers. In the case that no consensus could be reached, a third author (QX.L.) was consulted for a final decision.

Data from the studies fulfilled all selection criteria were extracted by one of the reviewers (L.C.). The other two reviewers (J.C. and Y.C.) verified the accuracy of the data. The data of outcomes were collected as the mean values and standard deviation. Besides, general characteristics data were extracted as follows: first author and publication year, study design, duration, number of patients and sites, age and gender of participants, and intervention. In situations where the required data were not available, the reviewers intended to contact the corresponding authors of the respective articles to obtain the missing information.

### Assessment of risk of bias

Two reviewers (L.C. and J.C.) evaluate the risk of bias based on the Cochrane Handbook for Systematic Reviews of Interventions [36]. Any disagreement was resolved by open discussion. Seven quality criteria were assessed: (1) random sequence generation (selection bias), (2) allocation concealment (selection bias), (3) blinding of participants and personnel (performance bias), (4) blinding of outcome assessment (detection bias), (5) incomplete outcome data (attrition bias), (6) selective outcome reporting (reporting bias), and (7) other bias.

Risk of bias in individual studies were classified as three categories: (1) low risk of bias: all seven criteria were at low risk of bias or six low risk of bias with only one unclear risk of bias; (2) moderate risk of bias: two or more criteria were at unclear risk of bias with no high risk of bias; (3) high risk of bias: one or more criteria were at high risk of bias. Heterogeneity across studies was assessed using Cochran-Q statistic and *I*^2^ statistic tests. Low heterogeneity was assigned with *I*^2^ values lower than 25%, moderate heterogeneity with values of 25–50%, and high heterogeneity with values of over 50% [38].

### Data analysis

To estimate the effect of intervention, continuous data from the included studies were reported as a mean difference (MD) and 95% confidence interval (CI). For studies with similar group comparisons, a meta-analysis was conducted, while a descriptive summary was provided for studies unavailable for meta-analysis. When there was good study homogeneity (*P* ≥ 0.10, I^2^ ≤ 50%), the fixed-effect model was applied to the meta-analysis. When high heterogeneity (*P* < 0.10, I^2^ > 50%) existed between the studies, the random-effects models were used. Data analysis were performed using Review Manager (RevMan version 5.4; The Cochrane Collaboration, Copenhagen, Denmark) [39].

## Results

### Study selection

**Fig. 1:**
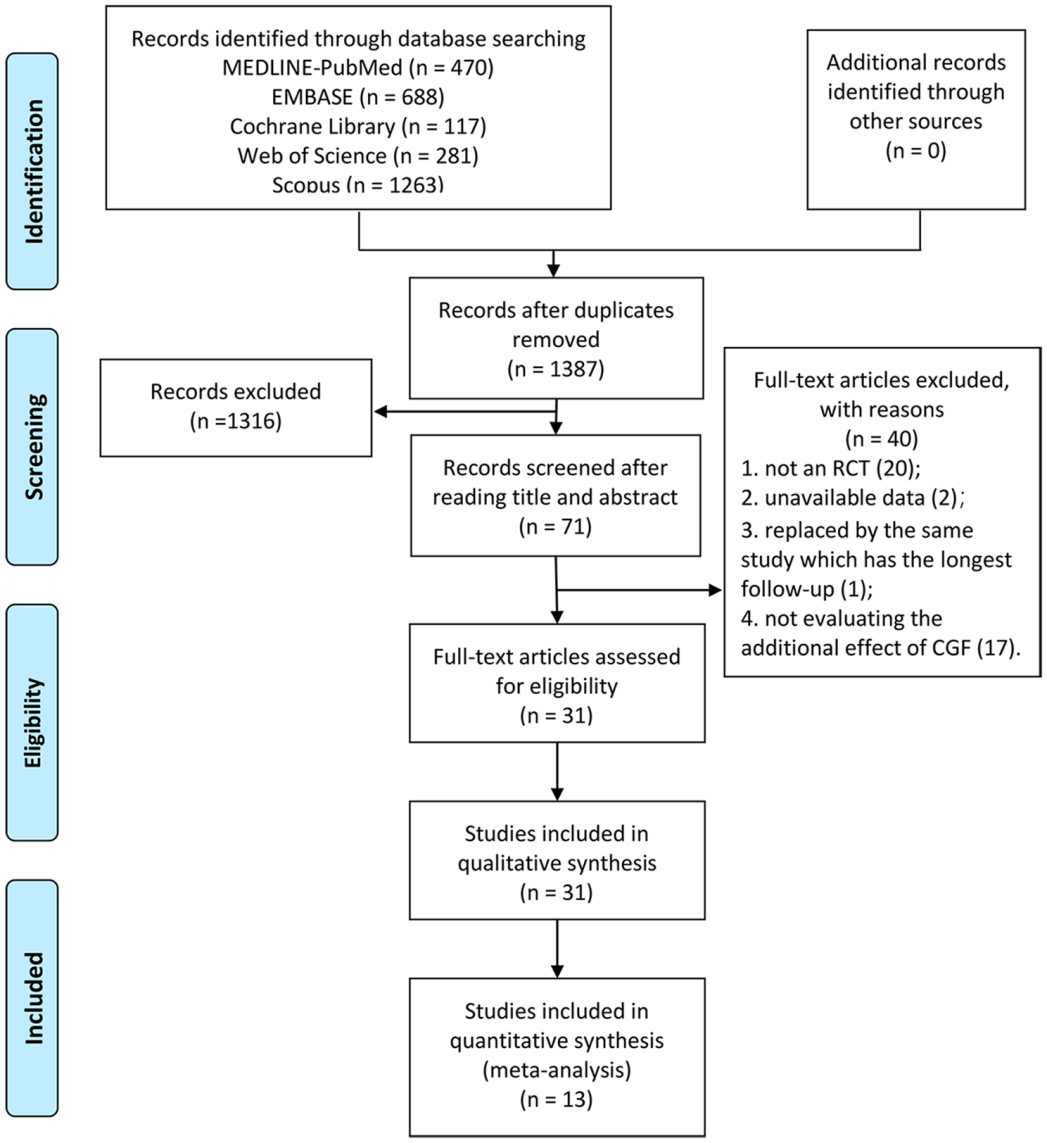
PRISMA flowchart for study selection

The flow diagram for selection process is shown in Fig. 1. As a result of the initial search, 2819 studies were obtained, of which 470 were found in PubMed, 688 in EMBASE, 117 in Cochrane Library, 281 in Web of Science, 1263 in Scopus and none in OpenGrey or Grey Literature Report. After duplicates removal, 1387 articles remained, which were further screened based on their titles and abstracts. Subsequently, 71 articles underwent a thorough assessment through a full-text review. Out of these, 31 RCTs fulfilled the inclusion criteria and were included for qualitative synthesis (main reason for exclusion were shown in Appendix 2). From the aforementioned 31 RCTs, 13 were eligible for inclusion in the meta-analysis.

### Assessment of risk of bias

**Fig. 2:**
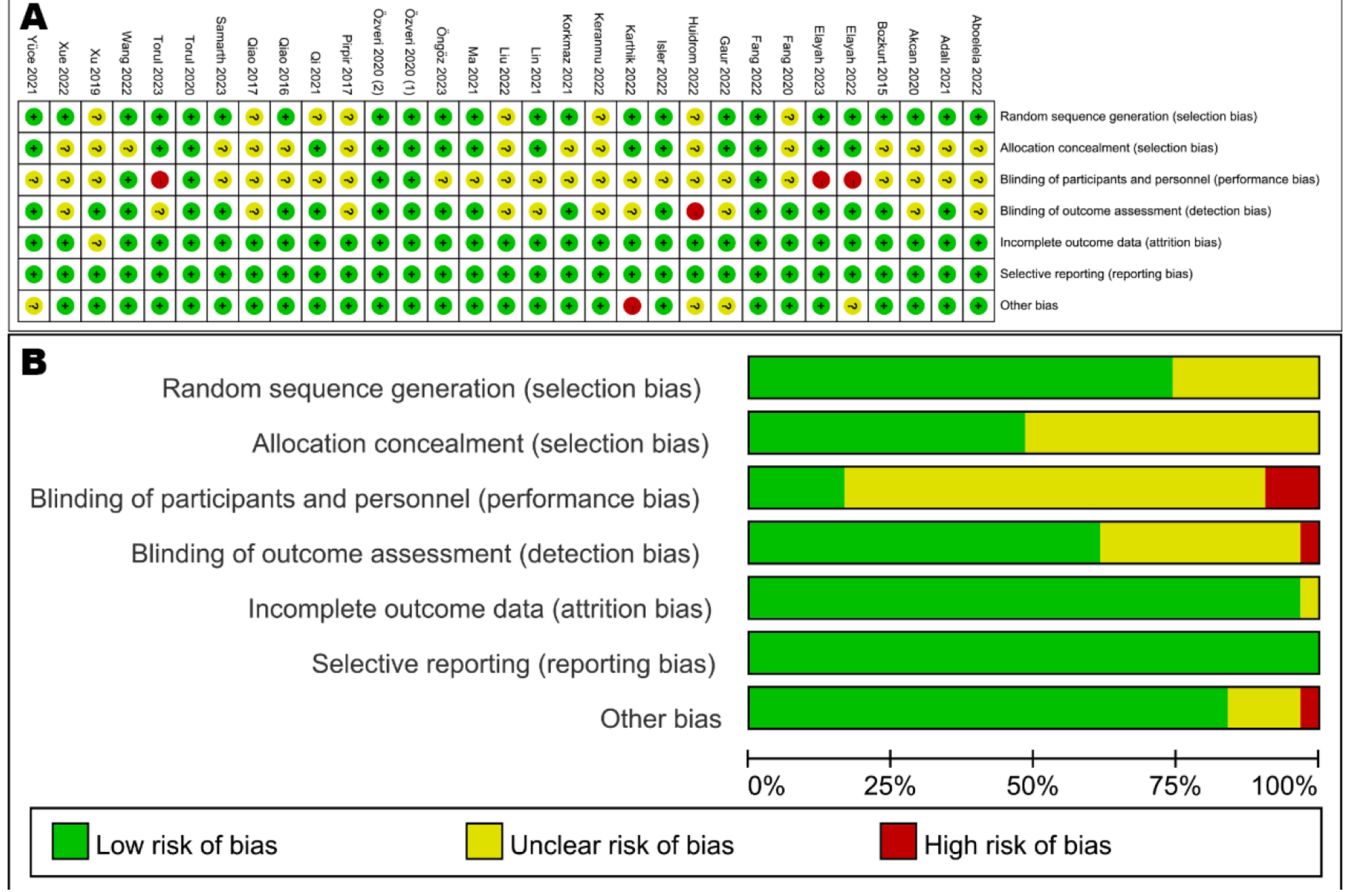
Risk of bias summary of the included studies

The risk of bias in selected 31 studies was summarized in Fig. 2. According to the Cochrane Collaboration’s tool for assessing the risk of bias, eight RCTs [40–47] were classified as having a low risk of bias, while five trials [48–52] presented a high risk of bias, and the other 18 trials [29, 53–69] demonstrated a moderate risk of bias (Fig. 2A). The main bias risks were selection bias, performance bias, and detection bias. More than 50% of trials displayed an unclear selection bias (allocation concealment), over 75% of trials had an unclear or high performance bias (blinding of participants and personnel), and about 40% of trials had an unclear or high detection bias (blinding of outcome assessment) (Fig. 2B).

### Synthesis of results

The 31 RCTs included were categorized based on various surgical treatments for different diseases. These included treatment of periodontal intrabony defects, treatment of gingival recession, treatment of furcation defects, treatment of implant-related diseases, tooth extraction of the third molar, alveolar ridge preservation, and treatment of other oral diseases. When there were two or more RCTs with the same intervention, a meta-analysis was conducted. For studies where meta-analysis was not feasible, only qualitative description was provided. In addition, a meta-analysis was performed to evaluate the effect of CGF on postoperative healing and pain after oral surgery.

#### Treatment of periodontal intrabony defects

**Fig. 3:**
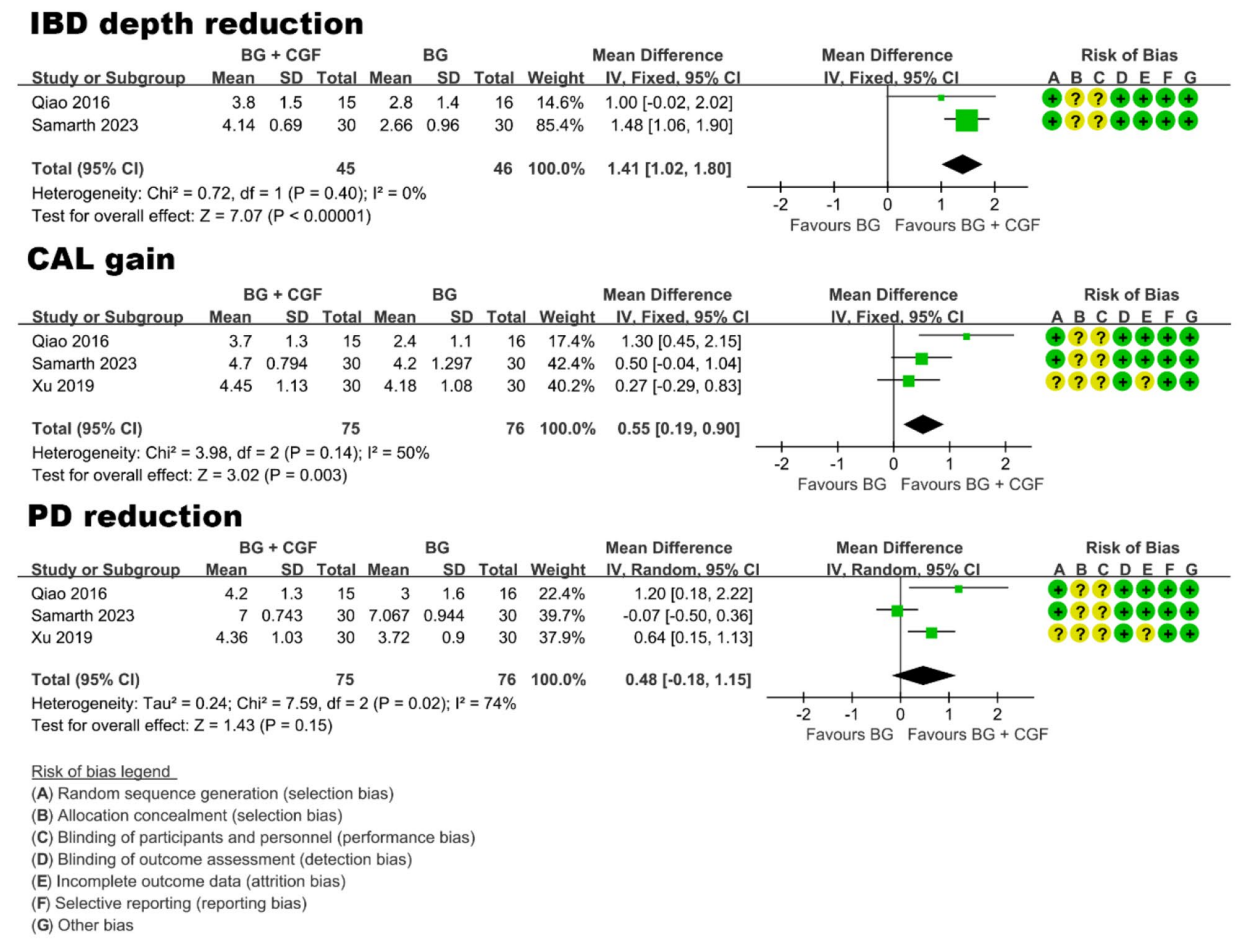
Forest plot of studies that evaluated IBD depth reduction, CAL gain, and PD reduction in the treatment of periodontal intrabony defects

Three studies [64, 66, 69] with a 12-month follow-up period reported the treatment of periodontal intrabony defects (Table 1). All three trials had a moderate risk of bias (Fig. 3). Only one study [66] compared the effects between open flap debridement (OFD) alone and OFD + CGF, and the result showed that OFD + CGF was significantly better than OFD alone in terms of PD reduction and CAL gain.

**Table 1:** Randomized trials reporting treatment of periodontal intrabony defects

Author (year)	Study Design, Blinded (duration)	Population			Intervention (number of surgical sites)		
		No. of participants (sites)	Age (mean/range)	gender		Control (sites)	Test (sites)
Qiao et al. (2016) [64]	Split-mouth and Parallel, examiner-blinded (12 months)	17 (31)	47.7 ± 13.9 (24–64)	7 M/10F	OFD + BO (16)		OFD + BO + CGF (15)
Xu et al. (2019) [66]	Parallel, examiner-blinded (12 months)	54 (120)	55.2 ± 8.3 (N)	32 M/26F	OFD (30)		T1: OFD + CGF (30); T2: OFD + BO (30); T3: OFD + BO + CGF (30)
Samarth et al. (2023) [69]	Split-mouth, examiner-blinded (12 months)	10 (60)	N (30–60)	3 M/7F	OFD + DFDBA (30)		T: OFD + DFDBA + CGF membrane (30)

OFD: open flap debridement; CGF: concentrated growth factor; BO: Bio-Oss®, Geistlich, anorganic bovine porous bone mineral for bone graft; DFDBA: demineralized freeze-dried bone allograft; N: not mentioned

Given that all three studies compared the effects of bone graft (BG) alone and BG combined with CGF (BG + CGF), a meta-analysis was conducted. Primary outcomes were intrabony defect (IBD) depth reduction and CAL gain, and secondary outcome was PD reduction. It is worth noting that only the study of Xu et al. [66] did not report data on IBD depth. The results of meta-analysis (Fig. 3) revealed that compared with BG alone, BG + CGF exhibited a statistically significant reduction in IBD depth, with a mean difference of 1.41 mm (95% CI: 1.02 to 1.80; P < 0.00001). Similarly, the BG + CGF group demonstrated a statistically significant beneficial effect on CAL gain, with a mean difference of 0.55 mm (95% CI: 0.19 to 0.90; P = 0.003). However, regarding PD reduction, no significant difference was observed between BG alone and BG + CGF (P > 0.05).

#### Treatment of gingival recession

**Fig. 4:**
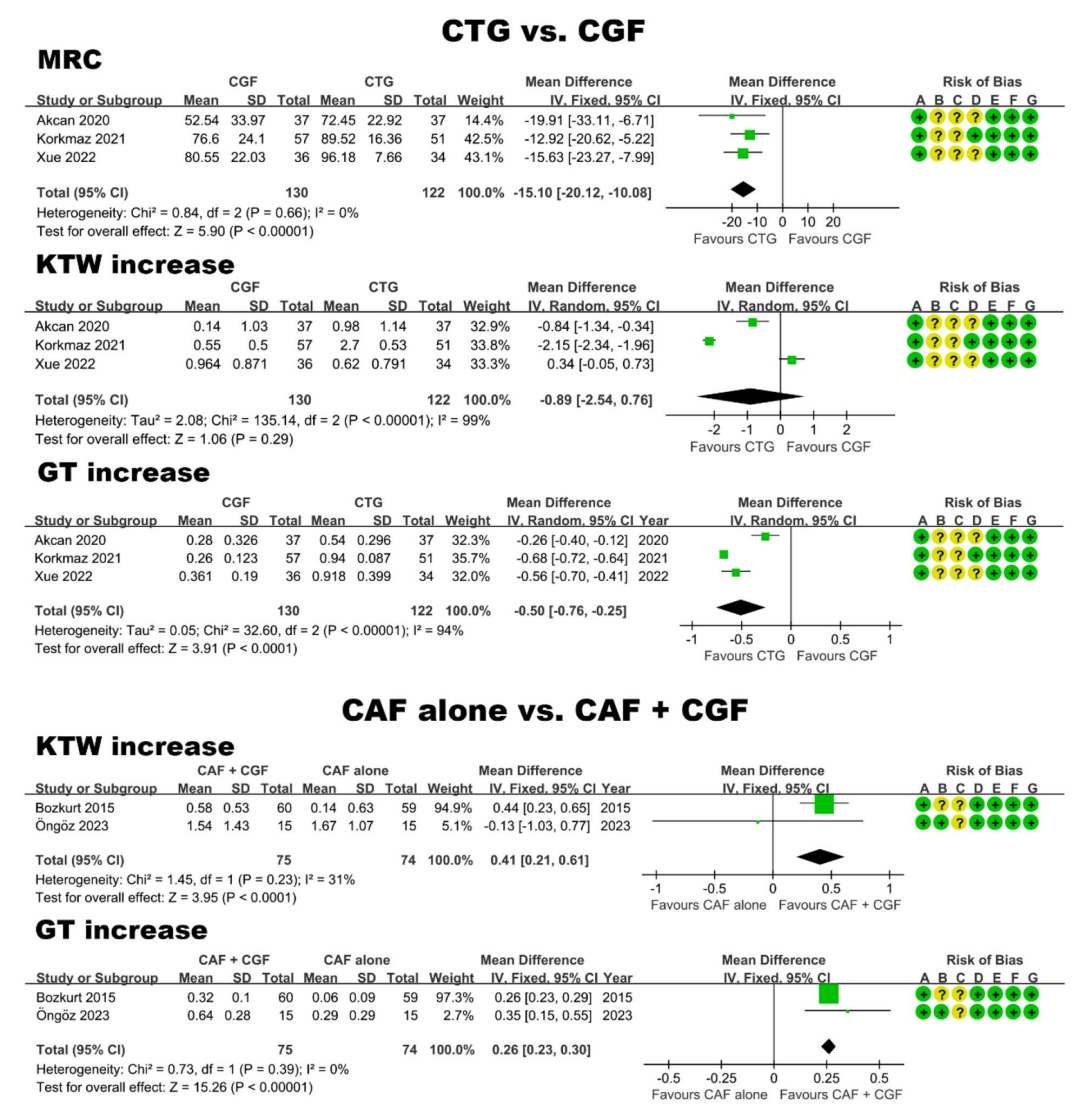
Forest plot of studies that evaluated MRC, KTW increase, and GT increase in the treatment of gingival recession

Five studies [29, 46, 55, 59, 67] reported the treatment of gingival recession (Table 2). All five included studies had a follow-up period of 6 months and enrolled participants with type Miller Class I and II gingival recession. Among these studies, four were categorized as having a moderate risk of bias, while one study [46] had a low risk of bias (Fig. 4). Within the included studies, two [29, 46] investigated the effects of coronally advanced flap (CAF) alone and CAF combined with CGF (CAF + CGF), while three studies [55, 59, 67] compared the therapeutic effects of connective tissue graft (CTG) and CGF as graft materials on gingival recession. Hence, two distinct comparisons were involved in the meta-analysis. Primary outcome was mean root coverage (MRC), and secondary outcomes were keratinized tissue width (KTW) increase and gingival thickness (GT) increase.

**Table 2:** Randomized trials reporting treatment of gingival recession

Author (year)	Study Design, Blinded (duration)	Population			Intervention (number of surgical sites)	
		No. of participants (sites)	Age (mean/range)	gender	Control (sites)	Test (sites)
Bozkurt et al. (2015) [29]	Split-mouth, examiner-blinded (6 months)	20 (119)	37.1 ± 1.03 (20–45)	7 M/13F	CAF (59)	CAF + CGF (60)
Akcan et al. (2020) [55]	Split-mouth, N (6 months)	19 (74)	N (20–63)	11 M/8F	CAF + CTG (37)	CAF + CGF (37)
Korkmaz et al. (2021) [59]	Parallel, examiner-blinded (6 months)	40 (108)	41.1 ± 9.3 (26–63)	N	TT + CTG (51)	TT + CGF (57)
Xue et al. (2022) [67]	Parallel, N (6 months)	28 (70)	38.56 ± 9.28 (N)	17 M/11F	CAT + CTG (34)	CAT + CGF (36)
Öngöz et al. (2023) [46]	Parallel, examiner-blinded (6 months)	16 (45)	35.31 ± 5.55 (25–45)	8 M/8F	CAF (15)	T1: CAF + CGF (15); T2: CAF + PRF (15)

CAF: coronally advanced flap; CTG: connective tissue graft; CGF: concentrated growth factor; TT: tunnel technique; N: not mentioned

**CTG vs. CGF.** As depicted in Fig. 4, MRC in the CTG group was significantly higher than that in the CGF group, showing a mean difference of 15.1% (95% CI: 10.08 to 20.12; P < 0.00001). Regarding GT increase, CTG demonstrated significant advantages over CGF, with a mean difference of 0.50 mm (95% CI: 0.25 to 0.76; P < 0.0001). However, there was no statistically significant difference between the two groups in terms of KTW increase (P > 0.05).

**CAF alone vs. CAF + CGF.** Due to the absence of standard deviation for the MRC data in one study [46], it was not possible to extract data and conduct a meta-analysis on this particular outcome. As shown in Fig. 4, CAF + CGF was found to significantly outperform CAF in terms of both KTW increase (mean difference: 0.41 mm; 95% CI: 0.21 to 0.61; P < 0.0001) and GT increase (mean difference: 0.26 mm; 95% CI: 0.23 to 0.30; P < 0.00001).

Furthermore, in addition to the treatment of gingival recession, one study [63] evaluated the effects of CGF on gingival thickness in patients with thin gingival phenotype undergoing periodontal accelerated osteogenic orthodontics (PAOO). The result of a parallel design RCT by Qi et al. [63], involving 240 anterior mandibular teeth, suggested that the use of CGF membrane in PAOO for patients with thin gingival phenotype could significantly increase gingival thickness compared with PAOO + collagen membrane.

#### Treatment of furcation defects

**Fig. 5:**
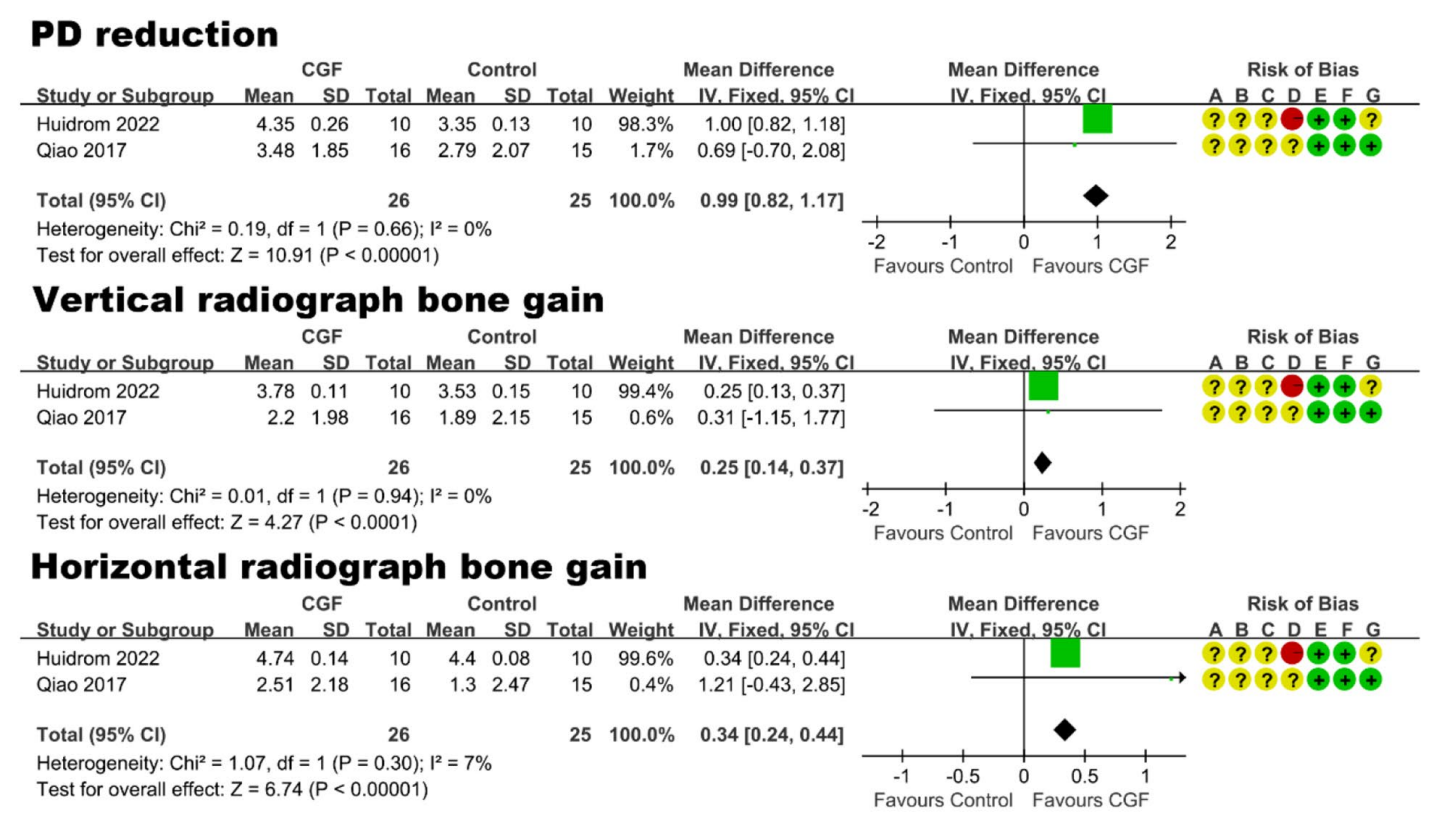
Forest plot of studies that evaluated PD reduction, vertical and horizontal radiograph bone gain in the treatment of furcation defects

Two studies [49, 65], containing 35 participants with 51 mandibular Class II furcation defects, reported the treatment of furcation defects. Follow-up periods ranged from 6 months to 12 months. In the RCT of Qiao et al. [65] with a moderate risk of bias (Fig. 5), the effect of CGF + BG and BG alone was evaluated. The study by Huidrom et al. [49] with a high risk of bias (Fig. 5) compared the effect of GTR + BG vs. GTR + BG + CGF. The result of meta-analysis (Fig. 5) showed that compared to control group without using CGF, a statistically significant effect of CGF in terms of PD reduction (mean difference: 0.99 mm, 95% CI: 0.82 to 1.17; P < 0.00001), vertical radiograph bone gain (mean difference: 0.25 mm, 95% CI: 0.14 to 0.37; P < 0.0001), and horizontal radiograph bone gain (mean difference: 0.34 mm, 95% CI: 0.24 to 0.44; P < 0.00001).

#### Treatment of implant-related diseases

Six RCTs [44, 50, 53, 54, 57, 62] reported the treatment of implant-related diseases (Table 3), including implant surgery, treatment of peri-implantitis, maxillary sinus lifting, and guided bone regeneration (GBR). Due to the diversity of diseases and the limited number of available articles, a qualitative description was provided instead of a meta-analysis.

**Table 3:** Randomized trials reporting alveolar ridge preservation

Author (year)	Study Design, Blinded (duration)	Population			Intervention (number of surgical sites)		
		No. of participants (sites)	Age (mean/range)	gender		Control (sites)	Test (sites)
Lin et al. (2021) [60]	Parallel, N (8 months)	36 (36)	48 (34–65)	21 M/15F	natural healing (12)		T1: BO + collagen membrane (12); T2: BO + CGF + CGF membrane (12)
Ma et al. (2021) [45]	Parallel, examiner-blinded (3 months)	46 (46)	43.98 ± 13.8 (23–72)	28 M/18F	natural healing (23)		CGF + CGF membrane (23)
Keranmu et al. (2022) [58]	Parallel, N (6 months)	38 (38)	28.89 ± 2.7 (N)	15 M/23F	BO + collagen membrane (19)		BO + CGF + CGF membrane + collagen membrane (19)
Liu et al. (2022) [61]	Parallel, N (6months)	22 (24)	30.46 ± 10.58 (19–61)	11 M/11F	BO + collagen membrane (12)		BO + CGF membrane (12)
Elayah et al. (2023) [51]	Split-mouth, examiner-blinded (3 months)	30 (60)	25 ± 0.5 (19–35)	16 M/14F	natural healing (30)		CGF (30)

**Implant surgery.** Karthik et al. [50] reported that when implants were placed with CGF compared to implants placed without CGF, a significantly higher bone density was observed at the first month, third month, and sixth month. Moreover, Pirpir et al. [62] reported that application of CGF seems to accelerate osseointegration of implants, which the implant stability quotient (ISQ) measurements at week 1 and week 4 were notably higher in the CGF group. However, concerning immediate dental implants, the study by Gaur et al. [57] revealed that CGF had no significant effect on the (ISQ, radiodensity, or the horizontal and vertical bone gap at 8, 12, or 16 weeks.

**Treatment of peri-implantitis.** Isler et al. [44] evaluated the effects of treating peri-implantitis by BG combined with collagen membrane or CGF membrane. After a 3-year follow-up period involving 51 patients, the changes in PD and radiographic vertical defect depth presented significantly greater reduction for BG + collagen membrane in comparison with BG + CGF membrane (p < 0.05). However, there were no significant differences between the two treatment modalities regarding treatment success outcomes.

**Maxillary sinus lifting.** Adalı et al. [54] conducted a split-mouth RCT involving 10 patients. The study compared maxillary sinus lifting combined with allograft mixed with CGF to maxillary sinus lifting combined with allograft alone. Cone-beam computed tomography analysis showed a significantly lower percentage of bone height resorption at the sixth month in the CGF group (median, 6.37%) compared to the allograft-alone group (median, 9.32%) (P < 0.05). However, the histomorphometric analysis revealed that there was no statistically significant difference between the two groups in the percentage of new bone formation.

**GBR.** Aboelela et al. [53] reported a parallel design RCT with 28 patients enrolled. The study compared the effect of collagen membrane and CGF membrane as barriers in GBR procedures. The control group received a GBR procedure using 1:1 mixture of particulate autogenous bone and xenograft, covered by collagen membrane, while the same graft materials were covered by CGF membrane in the test group. There was no statistically significant difference between CGF membrane and collagen membrane regarding bone gain.

#### Postoperative healing of tooth extraction

Five studies [40–43, 48] reported the effect of CGF on short-term clinical outcomes after mandibular third molar extraction, and one study [52] investigated the effect of CGF on the treatment of alveolar osteitis after tooth extraction. Table 4 provides a summary of these studies. The duration of follow-up varied, with five studies [41–43, 48, 52] assessing outcomes over a seven-day period and one study [40] observing patients for 24 weeks. In the studies focusing on healing after third molar extraction, CGF was applied to the extraction socket in the CGF group and natural healing after extraction was served as the control group. Due to the unavailability of data in the form of non-mean ± standard deviation values, meta-analysis could not be performed. Four studies [40–42, 48] reported favorable outcomes associated with CGF application, including reduced postoperative pain [40, 41, 48], swelling [41, 48], trismus [41], and incidence of alveolar osteitis [40, 42]. However, Torul et al. [43] demonstrated that CGF did not provide significant benefits in reducing pain, swelling, or trismus. Similarly, Fang et al. [40] reported no effect of CGF on reducing swelling and trismus. Regarding the treatment of alveolar osteitis, the study by Torul et al. [52] indicated that the combination of CGF and ozone provided faster and more satisfactory management of alveolar osteitis by effective pain and inflammation control and acceleration of healthy granulation tissue formation.

**Table 4:** Randomized trials reporting tooth extraction of third molar

Author (year)	Study Design, Blinded (duration)	Intervention (number of surgical sites)		Effects of CGF
		Control (sites)	Test (sites)	
Özveri et al. (2020) [42]	Split-mouth, patient and examiner-blinded (7 days)	tooth extraction (70)	tooth extraction + CGF (70)	Decreased the risk of alveolar osteitis development after mandibular third molar surgery.
Özveri et al. (2020) [41]	Split-mouth, patient and examiner-blinded (7 days)	tooth extraction (60)	tooth extraction + CGF (60)	Accelerated soft tissue healing and provided benefits in reducing postoperative pain, swelling and trismus on days 3 and 7.
Torul et al. (2020) [43]	Parallel, patient and examiner-blinded (7 days)	tooth extraction (25)	T1: tooth extraction + PRF (25); T2: tooth extraction + CGF (25)	Had no beneficial effect on pain (6th hour and 1st to 7th day), swelling (2nd and 7th days), and trismus (2nd and 7th days).
Elayah et al. (2022) [48]	Split-mouth, examiner-blinded (7 days)	tooth extraction (37)	tooth extraction + CGF (37)	Provided benefits in wound healing (7th day), swelling (1st and 3rd days), and pain (3rd and 7th days).
Fang et al. (2022) [40]	Parallel, patient and examiner-blinded (24 weeks)	tooth extraction (58)	tooth extraction + CGF (60)	Provided benefits in reducing postoperative pain (2, 24, and 48 h), reducing incidence of alveolar osteitis and increasing bone mineral density (24 weeks), but no benefit on swelling or trismus.
Torul et al. (2023) [52]	Parallel, examiner-blinded (7 days)	conventional treatment (25)	T1: ozone (20); T2: ozone + CGF (20)	Application of CGF and ozone together provided faster and more satisfactory management of alveolar osteitis.

CGF: concentrated growth factor; PRF: platelet-rich fibrin

#### Alveolar ridge preservation (ARP)

**Fig. 6:**
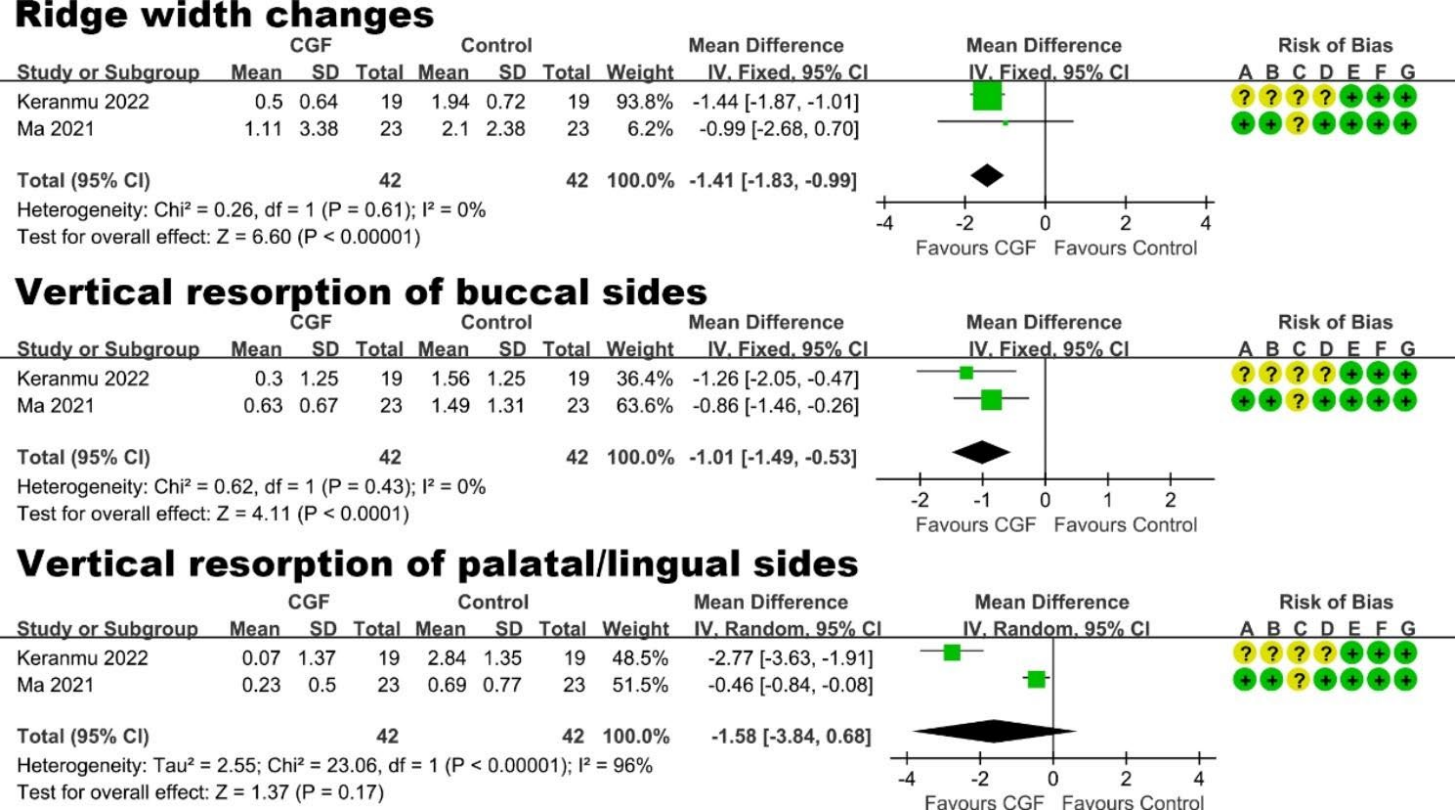
Forest plot of studies that evaluated ridge width changes of 3 mm below the alveolar bone crest, vertical resorption of buccal and palatal/lingual sides for ARP

Five studies [45, 51, 58, 60, 61] evaluated the efficacy of CGF in ARP procedures (Table 3). Follow-up periods ranged from 3 months to 8 months. Among them, one split-mouth RCT by Elayah et al. [51] reported that sockets grafted with CGF had better preservation of the alveolar ridge and PD reduction, compared to natural healing. Furthermore, two studies [45, 58] evaluated the additional effect of using both CGF clot and a CGF membrane (CGF + CGF membrane) in the ARP. The result of meta-analysis (Fig. 6) showed a statistically significant benefits of CGF + CGF membrane in the ARP operation in terms of ridge width changes of 3 mm below the alveolar bone crest (mean difference: -1.41 mm, 95% CI: -1.83 to -0.99 mm; P < 0.00001), and vertical resorption of buccal sides (mean difference: -1.01 mm, 95% CI: -1.49 to -0.53 mm; P < 0.0001).

The other two studies [60, 61] compared the effects of collagen membranes with CGF membrane as a barrier membrane. Liu et al. [61] reported that the application of CGF membrane had a faster rate of soft tissue healing and similar bone formation compared with Bio-Gide® collagen membranes. Furthermore, the result of study by Lin et al. [60] suggested that bone graft + CGF + CGF membrane effectively reduced the resorption of alveolar ridge and resulted in more newly formed bone than bone graft + collagen membrane.

BO: Bio-Oss®, Geistlich, anorganic bovine porous bone mineral for bone graft; CGF: concentrated growth factor; N: not mentioned.

#### Treatment of other oral diseases

Fang et al. [56] investigated the promoting effect of CGF on the repair of jaw bone defects. CGF combined with bone substitute was used to fill the jaw bone cavity in the test group, and bone substitute alone was used in the control group. The result demonstrated that bone mineral density in the bone defect area of the test group was significantly greater than that of the control group at 6 months postoperatively (P < 0.05). Yüce et al. [68] evaluated the efficiency of CGF on the healing process of osteoporotic patients with medication-related osteonecrosis of the jaws (MRONJ). After removal of necrotic bone, the surgical area was primarily closed with the additional use of CGF, which showed no statistically significant benefit than primarily closure alone in post-op healing data of MRONJ. Moreover, according to a split-mouth RCT [47], using CGF to wrap the mental nerve may accelerate the recovery of long-standing sensory nerve impairment following mental osteotomy.

#### Effects on postoperative healing and pain relief

**Fig. 7:**
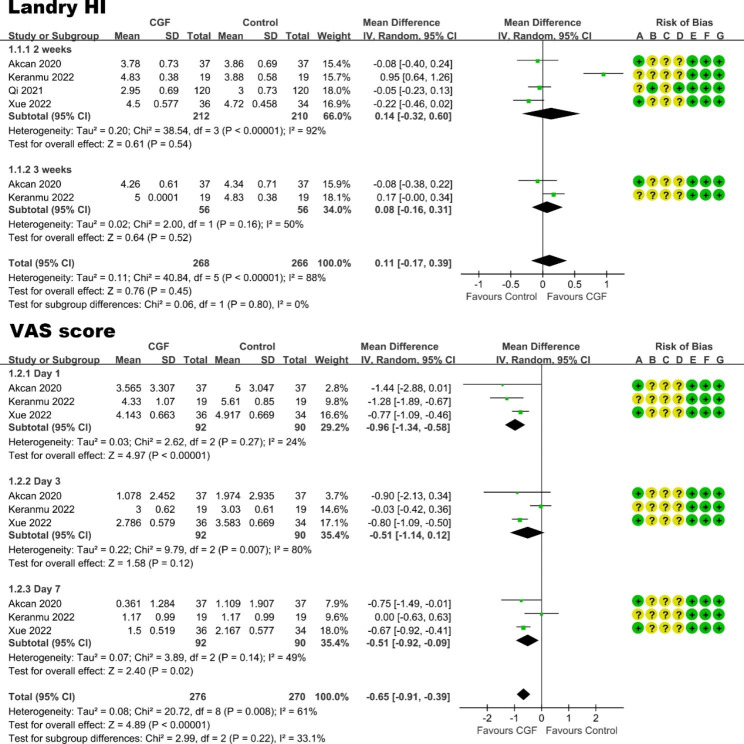
Forest plot of studies that evaluated Landry HI and VAS score for postoperative healing and pain

A meta-analysis was performed to evaluate the effect of CGF on postoperative healing and pain relief in oral surgery, and the Landry healing index (Landry HI) and VAS score were regarded as the outcomes. Due to limited reporting of patient-reported outcomes and variations in the timing of VAS and Landry HI results, only four [55, 58, 63, 67] out of the included 31 studies were suitable for meta-analysis. Among these studies, two [55, 67] were the treatment of gingival recession, one focused on the ARP, and one involved the PAOO procedure. In terms of Landry HI, based on the findings from meta-analysis (Fig. 7), the application of CGF did not significantly promote healing at 2 and 3 weeks after surgery. In terms of postoperative pain (VAS score), the result demonstrated that the VAS score of CGF group was significantly lower than that of the control group at the 1st and 7th day after oral surgery. In conclusion, when compared to the control group, the application of CGF resulted in a statistically positive effect on postoperative pain relief at 7 days after oral surgery, but had no statistically significant benefit on postoperative healing at 2 or 3 weeks.

## Discussion

### Summary of evidence

To the best of our knowledge, this is the first meta-analysis of CGF in the surgical treatment of oral diseases. The present systematic review and meta-analysis focused on evaluating the additional effect of CGF on enhancing hard and soft tissue healing in oral surgery. Grouping the included studies according to the type of oral diseases allowed us to reduce heterogeneity between studies and attempt a meta-analysis. While this systematic review identifies the potential positive effect of CGF in implant-related treatments, postoperative healing of tooth extraction, jaw defect reconstruction, and maxillofacial surgery, it is important to note that, presently, there is a lack of available meta-analysis data to substantiate these findings statistically. Herein, we summarised the key findings derived from the meta-analysis conducted as part of this study.

**Periodontal diseases.** Overall, CGF plays a significant positive role in the treatment of periodontal diseases. In the regenerative surgery of periodontal intrabony defects, BG + CGF was significantly superior to BG alone, in terms of mean IBD-depth-reduction mean of 1.41 mm (95% CI: 1.02 to 1.80; P < 0.00001) and mean CAL-gain difference of 0.55 mm (95% CI: 0.19 to 0.90; P = 0.003). In the regenerative surgery of furcation defects, the CGF group was significantly better than the control group, in terms of PD reduction (mean difference: 0.99 mm, 95% CI: 0.82 to 1.17; P < 0.00001), vertical radiographic bone gain (mean difference: 0.25 mm, 95% CI: 0.14 to 0.37; P < 0.0001), and horizontal radiographic bone gain (mean difference: 0.34 mm, 95% CI: 0.24 to 0.44; P < 0.00001). When it comes to surgical treatment for gingival recession, CTG was the gold standard graft material, surpassing CGF membrane graft significantly with a 15.1% difference (95% CI: 10.08 to 20.12; P < 0.00001) in MRC and a 0.50 mm difference (95% CI: 0.25 to 0.76; P < 0.0001) in GT increase. However, it is interesting to note that CAF + CGF outperforms CAF alone, showing significant differences in KTW increase (mean difference: 0.41 mm; 95% CI: 0.21 to 0.61; P < 0.0001) and GT increase (mean difference: 0.26 mm; 95% CI: 0.23 to 0.30; P < 0.00001).

**Alveolar ridge preservation (ARP).** The application of CGF provides potential advantages in the ARP procedure. Compared to natural healing or bone graft + collagen membrane, the additional use of CGF + CGF membrane may reduce horizontal bone resorption of 3 mm below the alveolar bone crest by 1.41 mm (95% CI: 0.99 to 1.83 mm; P < 0.00001) and reduce vertical bone resorption of buccal sides by 1.01 mm (95% CI: 0.53 to 1.49 mm; P < 0.0001).

**Effects on postoperative healing and pain.** Application of CGF in the oral surgery may have short-term benefits in terms of accelerating healing and pain relief within a week. Using Landry healing index and VAS score as the primary outcome variable, the result of meta-analysis showed that the application of CGF did not significantly promote healing at 2 and 3 weeks, but significantly promote postoperative pain relief at the 1st and 7th day after oral surgery.

In addition to the studies included in this review, we also noted the therapeutic potential of CGF in other oral diseases, such as regenerative endodontic procedures [70], autogenous tooth transplantation [71], treatment of dysplastic lesions of the oral mucosa [72], and treatment of temporomandibular disorders [73]. However, these articles primarily consist of retrospective studies or case reports, with a noticeable scarcity of RCTs. Despite this limitation, CGF demonstrates promising prospects for application in the treatment of oral diseases. CGF can promote the adhesion, proliferation, migration, and differentiation of a variety of cells, including periodontal ligament cells (PDLCs) [74, 75], stem cells from apical papilla [33, 75], dental stem pulp cells [76], and osteoblast cell [77]. Notably, CGF possesses antimicrobial and antibiofilm activity against *S. aureus* and *S. mutans* [78], which has important therapeutic implications in the oral cavity, where a large number of bacteria exist. Considering these findings, CGF exhibits promising prospects for application in the treatment of oral diseases by facilitating healing and regeneration of oral tissue.

### Various forms of CGF application

CGF can be utilized in various forms depending on the specific needs of the clinical situation. The most common forms of CGF application include its use as a clot, membrane, or combination with other biomaterials. CGF clots can continuously and steadily release growth factors for 14 days [19], can be used to promote wound healing, such as post-operative healing after tooth extraction. In the bone defect area, CGF is often employed in combination with bone graft materials, such as ARP, maxillary sinus lifting, jaw defects, periodontal intrabony defects, and furcation defects. When CGF is combined with bone graft materials, its growth factor release can be extended up to 28 days [79]. According to the histopathology observation in the study of Lin et al. [60], combined application of CGF and bone substitutes effectively resulted in more newly formed bone and decrease the percentage of residual materials. In the surgical treatment of gingival recession or GTR / GBR procedure, CGF is usually used in the form of a membrane. Our meta-analysis revealed an interesting result that CGF membrane transplantation can increase keratinized tissue width and gingival thickness, which may be that CGF promotes gingival regeneration through the AKT/Wnt/β-catenin and YAP signaling pathways [80]. CAF combined with CTG is regard as the gold standard treatment approach for gingival recession. When donor palatal mucosal tissue is insufficient, the use of CGF can be considered to increase the GT and KTW, which is cheaper than collagen membrane, while collagen membrane has no clear positive effect on the increase of gingival thickness [63, 81, 82].

In this systematic review, there were not enough RCTs to quantitatively compare the effects of CGF membranes with collagen membranes. Compared to collagen membrane, the application of CGF membrane provided a better soft tissue healing rate [61]. However, when the principle of guided tissue or bone regeneration (GTR or GBR) [44, 83] was applied to the treatment of periodontal intrabony defects [84], peri-implantitis [44, 85], or horizontal ridge augmentation [53], using a barrier membrane over the grafting material, CGF membrane did not provide superior clinical outcomes compared to collagen membranes. On the other hand, there is one RCT [60] that CGF combined with bone substitutes effectively reduced the resorption of alveolar ridge and resulted in more newly formed bone than collagen membranes combined with bone substitutes in the ARP procedure. Although CGF membrane has demonstrated potential clinical benefits, it is not a predictable barrier for GTR or GBR from a clinical perspective. Because the CGF membrane itself has a short resorption period of 2 weeks or less, it can barely maintain the sufficient space required for bone regeneration [53].

### Limitations

To adhere to high methodological standards and to maximize the clinical applicability of the results reported in this review, although stringent inclusion criteria were adopted, only eight studies in the included 31 RCTs was classified as a low risk of bias. Based on the assessment of risk of bias, more than 75% of the 31 included studies did not report whether patients were blinded, and more than 50% of the studies did not describe the process of allocation concealment, while over 40% of the studies did not provide information on blinding of outcome assessment. This common problem in the RCTs emphasizes the need for enhanced clarity in reporting blinding procedures and allocation concealment within RCT publications. In order to reduce bias of RCTs, the following protocol are recommended in the future studies: strictly recruit patients, use correct method of randomization and adequate allocation concealment, blinding of patients and outcome assessors, calibrate measurement results [13]. Moreover, only 13 of the 31 studies could be used for meta-analysis. Meta-analysis could not be performed for treatment of implant-related diseases, postoperative healing of tooth extraction, and treatment of other oral diseases due to various reasons. These include insufficient number of RCTs investigating the same intervention, variations in outcome measurement approaches, and unextractable data (e.g., non-mean ± standard deviation data). Because of the methodologic limitations of the existing studies, there is a need for further well-designed studies to provide more evidence in the precise role of CGF for oral surgery.

## Conclusions

In conclusion, this systematic review and meta-analysis have provided evidence supporting the beneficial effects of adjunctive use of CGF in the surgical treatment of oral diseases. The major findings can be summarized as follows:

1. CGF can be advantageously used as an adjunct to bone graft procedures for the treatment of periodontal intrabony defects and furcation defects.
2. CGF combined with CAF has a better therapeutic effect on gingival recession compared to CAF alone, although it is not as effective as CTG combined with CAF.
3. In the alveolar ridge preservation procedure, using CGF clot with a CGF membrane as an adjunct to natural healing or grafting procedures can reduce horizontal and vertical bone resorption.
4. The adjunctive application of CGF for the treatment of implant-related diseases, postoperative healing of tooth extraction, jaw defect reconstruction, and maxillofacial surgery, seems to have a positive clinical effect, whereas no data of meta-analysis supports it.
5. For the effects on postoperative healing and pain relief, CGF may promote healing and pain relief within a week in the oral surgery.
6. Compared to collagen membrane, CGF membrane may provide a better soft tissue healing rate and increase gingival thickness, while CGF membrane is not a predictable barrier for GTR or GBR from a clinical perspective.

### Electronic supplementary material

Below is the link to the electronic supplementary material.

Supplementary Material 1

## Data Availability

All data in this study are available within the manuscript.

## Abbreviations

CGF: Concentrated growth factor
BG: Bone graft
CAF: Coronally advanced flap
CTG: Connective tissue graft
GTR: Guided tissue regeneration
GBR: Guided bone regeneration
PRP: Platelet-rich plasma
PRF: Platelet-rich fibrin
RCTs: Randomized controlled trials
OFD: Open-flap debridement
CAL: Clinical attachment level
PD: Probing depth
MRC: Mean root coverage
KTW: Keratinized tissue width
GT: Gingival thickness
PAOO: Periodontal accelerated osteogenic orthodontics
MD: Mean difference
CI: Confidence interval

## References

[1] Peres MA, Macpherson LMD, Weyant RJ, Daly B, Venturelli R, Mathur MR (2019). Oral diseases: a global public health challenge. Lancet (London England).

[2] Sculean A, Nikolidakis D, Nikou G, Ivanovic A, Chapple IL, Stavropoulos A (2015). Biomaterials for promoting periodontal regeneration in human intrabony defects: a systematic review. Periodontol 2000.

[3] Haugen HJ, Lyngstadaas SP, Rossi F, Perale G (2019). Bone grafts: which is the ideal biomaterial?. J Clin Periodontol.

[4] Al-Hamed FS, Mahri M, Al-Waeli H, Torres J, Badran Z, Tamimi F (2019). Regenerative effect of platelet concentrates in oral and Craniofacial Regeneration. Front Cardiovasc Med.

[5] Del Fabbro M, Karanxha L, Panda S, Bucchi C, Nadathur Doraiswamy J, Sankari M (2018). Autologous platelet concentrates for treating periodontal infrabony defects. Cochrane Database Syst Rev.

[6] Whitman DH, Berry RL, Green DM (1997). Platelet gel: an autologous alternative to fibrin glue with applications in oral and maxillofacial surgery. J oral Maxillofacial Surgery: Official J Am Association Oral Maxillofacial Surg.

[7] Dohan Ehrenfest DM, Rasmusson L, Albrektsson T (2009). Classification of platelet concentrates: from pure platelet-rich plasma (P-PRP) to leucocyte- and platelet-rich fibrin (L-PRF). Trends Biotechnol.

[8] Piccin A, Di Pierro AM, Canzian L, Primerano M, Corvetta D, Negri G (2017). Platelet gel: a new therapeutic tool with great potential. Blood Transfus.

[9] Choukroun J, Adda F, Schoeffer C, Vervelle A (2000). PRF: an opportunity in perio-implantology. Implantodontie.

[10] Kobayashi M, Kawase T, Okuda K, Wolff LF, Yoshie H (2015). In vitro immunological and biological evaluations of the angiogenic potential of platelet-rich fibrin preparations: a standardized comparison with PRP preparations. Int J Implant Dent.

[11] Castro AB, Meschi N, Temmerman A, Pinto N, Lambrechts P, Teughels W (2017). Regenerative potential of leucocyte- and platelet-rich fibrin. Part A: intra-bony defects, furcation defects and periodontal plastic surgery. A systematic review and meta-analysis. J Clin Periodontol.

[12] Castro AB, Meschi N, Temmerman A, Pinto N, Lambrechts P, Teughels W (2017). Regenerative potential of leucocyte- and platelet-rich fibrin. Part B: sinus floor elevation, alveolar ridge preservation and implant therapy. A systematic review. J Clin Periodontol.

[13] Chen L, Ding Y, Cheng G, Meng S (2021). Use of platelet-rich fibrin in the treatment of Periodontal Intrabony defects: a systematic review and Meta-analysis. BioMed Res Int Journal Translated Name BioMed Research International.

[14] Tarallo F, Mancini L, Pitzurra L, Bizzarro S, Tepedino M, Marchetti E. Use of platelet-rich fibrin in the treatment of Grade 2 Furcation defects: systematic review and Meta-analysis. J Clin Med. 2020;9(7). 10.3390/jcm9072104 PMC7408882 32635413

[15] Rodella LF, Favero G, Boninsegna R, Buffoli B, Labanca M, Scarì G (2011). Growth factors, CD34 positive cells, and fibrin network analysis in concentrated growth factors fraction. Microsc Res Tech.

[16] Sacco L, Lecture (2006). International academy of implant prosthesis and osteoconnection. Lecture.

[17] Isobe K, Watanebe T, Kawabata H, Kitamura Y, Okudera T, Okudera H (2017). Mechanical and degradation properties of advanced platelet-rich fibrin (A-PRF), concentrated growth factors (CGF), and platelet-poor plasma-derived fibrin (PPTF). Int J Implant Dent.

[18] Masuki H, Okudera T, Watanebe T, Suzuki M, Nishiyama K, Okudera H (2016). Growth factor and pro-inflammatory cytokine contents in platelet-rich plasma (PRP), plasma rich in growth factors (PRGF), advanced platelet-rich fibrin (A-PRF), and concentrated growth factors (CGF). Int J Implant Dent.

[19] Lei L, Yu Y, Han J, Shi D, Sun W, Zhang D (2020). Quantification of growth factors in advanced platelet-rich fibrin and concentrated growth factors and their clinical efficacy as adjunctive to the GTR procedure in periodontal intrabony defects. J Periodontol.

[20] Lee HM, Shen EC, Shen JT, Fu E, Chiu HC, Hsia YJ (2020). Tensile strength, growth factor content and proliferation activities for two platelet concentrates of platelet-rich fibrin and concentrated growth factor. J Dent Sci.

[21] Li S, Yang H, Duan Q, Bao H, Li A, Li W (2022). A comparative study of the effects of platelet-rich fibrin, concentrated growth factor and platelet-poor plasma on the healing of tooth extraction sockets in rabbits. BMC Oral Health.

[22] Hu Y, Jiang Y, Wang M, Tian W, Wang H (2018). Concentrated growth factor enhanced Fat Graft Survival: a comparative study. Dermatol Surg.

[23] Aghamohamadi Z, Kadkhodazadeh M, Torshabi M, Tabatabaei F (2020). A compound of concentrated growth factor and periodontal ligament stem cell-derived conditioned medium. Tissue Cell.

[24] Hong S, Chen W, Jiang B (2018). A comparative evaluation of concentrated growth factor and platelet-rich fibrin on the Proliferation, Migration, and differentiation of human stem cells of the apical papilla. J Endod.

[25] Jun H, Lei D, Qifang Y, Yuan X, Deqin Y (2018). Effects of concentrated growth factors on the angiogenic properties of dental pulp cells and endothelial cells: an in vitro study. Braz Oral Res.

[26] Chen X, Wang J, Yu L, Zhou J, Zheng D, Zhang B (2018). Effect of concentrated growth factor (CGF) on the Promotion of Osteogenesis in Bone Marrow Stromal cells (BMSC) in vivo. Sci Rep.

[27] Yin X, Shi H, Hze-Khoong EP, Hu Y, Zhang C (2022). Effect of concentrated growth factor on Distraction Osteogenesis of Dental Implant Distractors. J oral Maxillofacial Surgery: Official J Am Association Oral Maxillofacial Surg.

[28] Chen H, Zhou L, Wu D, Zhang J, Zheng Y, Chen Y (2022). Osteotome sinus floor elevation with concentrated growth factor and simultaneous implant placement with or without bone grafting: a retrospective study. Int J Oral Maxillofac Surg.

[29] Bozkurt Dogan S, Ongoz Dede F, Balli U, Atalay EN, Durmuslar MC (2015). Concentrated growth factor in the treatment of adjacent multiple gingival recessions: a split-mouth randomized clinical trial. J Clin Periodontol.

[30] Wang F, Sun Y, He D, Wang L (2017). Effect of concentrated growth factors on the repair of the Goat Temporomandibular Joint. J oral Maxillofacial Surgery: Official J Am Association Oral Maxillofacial Surg.

[31] Chen J, Jiang H (2020). A comprehensive review of concentrated growth factors and their novel applications in facial reconstructive and regenerative medicine. Aesthetic Plast Surg.

[32] Chen J, Wan Y, Lin Y, Jiang H (2021). Considerations for clinical use of concentrated growth factor in Maxillofacial Regenerative Medicine. J Craniofac Surg.

[33] Hong S, Li L, Cai W, Jiang B (2019). The potential application of concentrated growth factor in regenerative endodontics. Int Endod J.

[34] Li Z, Liu L, Wang L, Song D (2021). The effects and potential applications of concentrated growth factor in dentin-pulp complex regeneration. Stem Cell Res Ther.

[35] Lokwani BV, Gupta D, Agrawal RS, Mehta S, Nirmal NJ (2020). The use of concentrated growth factor in dental implantology: a systematic review. J Indian Prosthodont Soc.

[36] Higgins JPTTJ, Chandler J, Cumpston M, Li T, Page MJ, Welch VA, editors. Cochrane Handbook for Systematic Reviews of Interventions. 2nd Edition. ed. Chichester (UK): John Wiley & Sons; 2019.

[37] Moher D, Liberati A, Tetzlaff J, Altman DG (2009). Preferred reporting items for systematic reviews and meta-analyses: the PRISMA statement. PLoS Med.

[38] Higgins JP, Thompson SG, Deeks JJ, Altman DG (2003). Measuring inconsistency in meta-analyses. BMJ.

[39] Review Manager (RevMan). [Computer program]. Version 5.4. The Cochrane Collaboration; 2020.

[40] Fang D, Li D, Li C, Yang W, Xiao F, Long Z (2022). Efficacy and safety of concentrated growth factor fibrin on the extraction of Mandibular Third Molars: a prospective, randomized, double-blind controlled clinical study. J oral Maxillofacial Surgery: Official J Am Association Oral Maxillofacial Surg.

[41] Özveri Koyuncu B, Işık G, Özden Yüce M, Günbay S, Günbay T (2020). Effect of concentrated growth factor (CGF) on short-term clinical outcomes after partially impacted mandibular third molar surgery: a split-mouth randomized clinical study. J Stomatol Oral Maxillofac Surg.

[42] Özveri Koyuncu B, Işık G, Özden Yüce M, Günbay S, Günbay T (2020). Effect of concentrated growth factors on frequency of alveolar osteitis following partially-erupted mandibular third molar surgery: a randomized controlled clinical study. BMC Oral Health.

[43] Torul D, Omezli MM, Kahveci K. Evaluation of the effects of concentrated growth factors or advanced platelet rich-fibrin on postoperative pain, edema, and trismus following lower third molar removal: a randomized controlled clinical trial. J Stomatol Oral Maxillofac Surg. 2020. 10.1016/j.jormas.2020.02.004 32068167

[44] Isler SC, Soysal F, Ceyhanlı T, Bakırarar B, Unsal B. Efficacy of concentrated growth factor versus collagen membrane in reconstructive surgical therapy of peri-implantitis: 3-year results of a randomized clinical trial. Clin Oral Invest. 2022. 10.1007/s00784-022-04493-y PMC9381616 35618961

[45] Ma F, Lin Y, Sun F, Jiang X, Wei T (2021). The impact of autologous concentrated growth factors on the alveolar ridge preservation after posterior tooth extraction: a prospective, randomized controlled clinical trial. Clin Implant Dent Relat Res.

[46] Öngöz Dede F, Bozkurt Doğan Ş, Çelen K, Çelen S, Deveci ET, Seyhan Cezairli N (2023). Comparison of the clinical efficacy of concentrated growth factor and advanced platelet-rich fibrin in the treatment of type I multiple gingival recessions: a controlled randomized clinical trial. Clin Oral Invest.

[47] Wang X, Chen XP, Zhao QM, Huang XX, Wang XW, Long XH (2022). Effect of concentrated growth factor on lower lip hypoesthesia after osseous genioplasty: a prospective, split-mouth, double-blind randomized controlled trial. Int J Oral Maxillofac Surg.

[48] Elayah SA, Liang X, Sakran KA, Xie L, Younis H, Alajami AE (2022). Effect of concentrated growth factor (CGF) on postoperative sequel of completely impacted lower third molar extraction: a randomized controlled clinical study. BMC Oral Health.

[49] Huidrom E, Srivastava V, Meenawat A, Srivastava A, Khan YS, Shahni R (2022). Evaluation of the efficacy of concentrated growth factor along with bovine-derived xenograft and collagen membrane in the treatment of degree II mandibular molar furcation defect - A clinicoradiographic study. J Indian Soc Periodontology.

[50] Karthik VC, Prabhu K, Bharath N, Shobana P, Indhu K, Abraham S (2022). Randomized controlled study on effect of concentrated growth factors on crestal bone levels and peri-implant bone density in dental implants. J Pharm Bioallied Sci.

[51] Elayah SA, Younis H, Cui H, Liang X, Sakran KA, Alkadasi B (2023). Alveolar ridge preservation in post-extraction sockets using concentrated growth factors: a split-mouth, randomized, controlled clinical trial. Front Endocrinol (Lausanne).

[52] Torul D, Omezli MM, Avci T. Investigation of the clinical efficacy of CGF and ozone in the management of alveolar osteitis: a randomized controlled trial. Clin Oral Invest. 2023. 10.1007/s00784-023-05074-3 37231273

[53] Aboelela SAA, Atef M, Shawky M, Fattouh H. Ridge augmentation using autologous concentrated growth factors enriched bone graft matrix versus guided bone regeneration using native collagen membrane in horizontally deficient maxilla: A randomized clinical trial. Clinical implant dentistry and related research. 2022. 10.1111/cid.13121 35811435

[54] Adalı E, Yüce MO, Günbay T, Günbay S (2021). Does concentrated growth factor used with Allografts in Maxillary Sinus Lifting have adjunctive benefits?. J oral Maxillofacial Surgery: Official J Am Association Oral Maxillofacial Surg.

[55] Akcan SK, Ünsal B (2020). Gingival recession treatment with concentrated growth factor membrane: a comparative clinical trial. J Appl oral Science: Revista FOB.

[56] Fang D, Long Z, Hou J (2020). Clinical application of concentrated growth factor fibrin combined with bone repair materials in Jaw defects. J oral Maxillofacial Surgery: Official J Am Association Oral Maxillofacial Surg.

[57] Gaur S, Chugh A, Chaudhry K, Bajpayee A, Jain G, Chugh VK (2022). Efficacy and safety of concentrated growth factors and Platelet- Rich Fibrin on Stability and Bone Regeneration in patients with Immediate Dental Implants: a Randomized Controlled Trial. Int J Oral Maxillofac Implants.

[58] Keranmu D, Nuermuhanmode N, Ainiwaer A, Guli, Taxifulati D, Shan W (2022). Clinical application of concentrate growth factors combined with bone substitute in alveolar ridge preservation of anterior teeth. BMC Oral Health.

[59] Korkmaz B, Balli U (2021). Clinical evaluation of the treatment of multiple gingival recessions with connective tissue graft or concentrated growth factor using tunnel technique: a randomized controlled clinical trial. Clin Oral Invest.

[60] Lin SC, Li X, Liu H, Wu F, Yang L, Su Y (2021). Clinical applications of concentrated growth factors combined with bone substitutes for alveolar ridge preservation in maxillary molar area: a randomized controlled trial. Int J Implant Dent.

[61] Liu Y, Li X, Jiang C, Guo H, Luo G, Huang Y (2022). Clinical applications of concentrated growth factors membrane for sealing the socket in alveolar ridge preservation: a randomized controlled trial. Int J Implant Dent.

[62] Pirpir C, Yilmaz O, Candirli C, Balaban E (2017). Evaluation of effectiveness of concentrated growth factor on osseointegration. Int J Implant Dent.

[63] Qi L, Ge W, Cao N, Wang S, Qian Y, Wang X (2021). Effects of autologous concentrated growth factor on gingival thickness in periodontal accelerated osteogenic orthodontics: a 6-month randomized controlled trial. BMC Oral Health.

[64] Qiao J, Duan J, Zhang Y, Chu Y, Sun C (2016). The effect of concentrated growth factors in the treatment of periodontal intrabony defects. Futur Sci OA.

[65] Qiao J, Duan JY, Chu Y, Sun CZ (2017). Effect of concentrated growth factors on the treatment of degree II furcation involvements of mandibular molars. Beijing Da Xue Xue Bao Yi Xue Ban.

[66] Xu Y, Qiu J, Sun Q, Yan S, Wang W, Yang P (2019). One-year results evaluating the Effects of concentrated growth factors on the Healing of Intrabony defects treated with or without bone substitute in Chronic Periodontitis. Med Sci Monitor: Int Med J Experimental Clin Res.

[67] Xue F, Zhang R, Zhang Y, Liu J, Cai Y, Cao P (2022). Treatment of multiple gingival recessions with concentrated growth factor membrane and coronally advanced tunnel technique via digital measurements: a randomized controlled clinical trial. J Dent Sci.

[68] Yüce MO, Adalı E, Işık G (2021). The effect of concentrated growth factor (CGF) in the surgical treatment of medication-related osteonecrosis of the jaw (MRONJ) in osteoporosis patients: a randomized controlled study. Clin Oral Invest.

[69] Samarth G, Kolte A, Kolte R, Bajaj V (2023). Comparative evaluation of demineralized freeze-dried bone allograft with and without concentrated growth factor membrane in the treatment of periodontal intrabony defects: a randomized controlled clinical trial. Clin Oral Invest.

[70] Cheng J, Yang F, Li J, Hua F, He M, Song G (2022). Treatment outcomes of regenerative endodontic procedures in traumatized immature permanent necrotic teeth: a retrospective study. J Endod.

[71] Keranmu D, Ainiwaer A, Nuermuhanmode N, Ling W (2021). Application of concentrated growth factor to autotransplantation with inflammation in recipient area. BMC Oral Health.

[72] Lin SL, Wu SL, Tsai CC, Ko SY, Chiang WF, Yang JW (2016). The use of solid-phase concentrated growth factors for Surgical defects in the treatment of dysplastic lesions of the oral mucosa. J Oral Maxillofac Surg Journal Translated Name Journal of Oral and Maxillofacial Surgery.

[73] Yang JW, Huang YC, Wu SL, Ko SY, Tsai CC. Clinical efficacy of a centric relation occlusal splint and intra-articular liquid phase concentrated growth factor injection for the treatment of temporomandibular disorders. Med J Translated Name Med. 2017;96(11). 10.1097/MD.0000000000006302 PMC5369893 28296738

[74] Zhan X, Yan W, Yan J, Tong W, Chen W, Lin Y (2021). LPCGF and EDTA conditioning of the root surface promotes the adhesion, growth, migration and differentiation of periodontal ligament cells. J Periodontol.

[75] Yang F, Zhang R, Xu J, Du J, Leng S, Zhang L et al. Comparative Effects of concentrated growth factors on the Biological characteristics of Periodontal Ligament cells and stem cells from apical papilla. J Endod. 2022. 10.1016/j.joen.2022.05.001 35545146

[76] Xu F, Qiao L, Zhao Y, Chen W, Hong S, Pan J (2019). The potential application of concentrated growth factor in pulp regeneration: an in vitro and in vivo study. Stem Cell Res Ther.

[77] Sahin IO, Gokmenoglu C, Kara C. Effect of concentrated growth factor on osteoblast cell response. J Stomatol Oral Maxillofac Surg. 2018. 10.1016/j.jormas.2018.06.001 29885910

[78] Alauddin MS, Yusof NM, Adnan AS, Said Z (2022). Preliminary novel analysis on Antimicrobial Properties of concentrated growth factor against Bacteria-Induced oral Diseases. Eur J Dent.

[79] Yu M, Wang X, Liu Y, Qiao J (2019). Cytokine release kinetics of concentrated growth factors in different scaffolds. Clin Oral Invest.

[80] Qi L, Liu L, Hu Y, Li J, Li J, Cao N (2020). Concentrated growth factor promotes gingival regeneration through the AKT/Wnt/β-catenin and YAP signaling pathways. Artif Cells Nanomed Biotechnol.

[81] Dandu SR, Murthy KR (2016). Multiple gingival recession defects treated with Coronally Advanced Flap and either the VISTA technique enhanced with GEM 21S or Periosteal Pedicle Graft: a 9-Month Clinical Study. Int J Periodontics Restor Dent.

[82] Sanz-Martin I, Ferrantino L, Vignoletti F, Nuñez J, Baldini N, Duvina M (2018). Contour changes after guided bone regeneration of large non-contained mandibular buccal bone defects using deproteinized bovine bone mineral and a porcine-derived collagen membrane: an experimental in vivo investigation. Clin Oral Invest.

[83] Sheikh Z, Qureshi J, Alshahrani AM, Nassar H, Ikeda Y, Glogauer M (2017). Collagen based barrier membranes for periodontal guided bone regeneration applications. Odontology.

[84] Shoukheba MYM, ELkholy SE, Badr AM (2021). Concentrated growth factors membrane in the treatment of Intrabony Periodontal defects in localized aggressive periodontitis. A Randomized Controlled Split-Mouth Clinical Study. Teikyo Med J.

[85] Isler SC, Soysal F, Ceyhanli T, Bakirarar B, Unsal B (2018). Regenerative surgical treatment of peri-implantitis using either a collagen membrane or concentrated growth factor: a 12-month randomized clinical trial. Clin Implant Dent Relat Res.

