# Supplementary material for: Efficacy of concentrated growth factor (CGF) in the surgical treatment of oral diseases: a systematic review and meta-analysis
Source: BMC Oral Health. 2023 Oct 4;23:712. doi: 10.1186/s12903-023-03357-5 (PMC10548564; doi:10.1186/s12903-023-03357-5)
Supplement: Supplementary file 1 — Supplementary Material 1 [file 12903_2023_3357_MOESM1_ESM.docx]

**Appendix**

**Appendix 1: search strategy**

**1.1 MEDLINE-PubMed**

#1 Search: ((CGF) OR (concentrated growth factor[MeSH Terms])) AND ((((((((((((((periodontal disease[MeSH Terms]) OR (gingival)) OR (furcation defect[MeSH Terms])) OR (periodontitis[MeSH Terms])) OR (dental implant[MeSH Terms])) OR (periimplantitis[MeSH Terms])) OR (periapical disease[MeSH Terms])) OR (maxillary)) OR (jawbone defect)) OR (tooth extraction[MeSH Terms])) OR (alveolar ridge preservation[MeSH Terms])) OR (tooth)) OR (dental)) OR (maxillofacial)) Filters: Clinical Study, Clinical Trial, Meta-Analysis, Randomized Controlled Trial, Review, Systematic Review, Humans, from 2006 – 2023 (470)

**1.2 EMBASE**

No. Query Results Results

#3. #1 OR #2 688

#2. 'concentrated growth factor'/exp OR 'concentrated growth factor' 212

#1. cgf 631

**1.3 Cochrane Library**

ID Search Hits

#1 concentrated growth factor in Trials 117

**1.4 Web of science**

#1 concentrated growth factor (Topic) OR CGF (Topic) AND Clinical Trial (Document Types) (281)

**1.5 Scopus**

#1 TITLE-ABS-KEY ( concentrated AND growth AND factor ) AND PUBYEAR > 2006 AND PUBYEAR < 2024 AND ( LIMIT-TO ( SUBJAREA , "MEDI" ) OR LIMIT-TO ( SUBJAREA , "DENT" ) ) (1263)

**Appendix 2: Full-text articles excluded, with reasons**

| Main reason for exclusion | Study |
| --- | --- |
| not an RCT (n=20) | Chen et al. (2022), Lei et al. (2020), Sohn et al. (2011), Park et al. (2011), Kim et al. (2014), Chen et al. (2016), Dai et al. (2020), Taschieri et al. (2021), Wang et al. (2021), Chen et al. (2022), Merli et al. (2022), Shetty (2018), Manoj (2018), Kamal et al. (2020) 1, Kamal et al. (2020) 2, Doan et al. (2019), Yang et al. (2017), Lin et al. (2016), Keranmu (2021), Shetye (2022) |
| replaced by the sanme study which has the longest follow-up. (n=1) | Isler et al. (2018) |
| unavailable data (n=2) | Kabilamurthi (2021), Gnatek (2019) |
| not evaluating the additional effect of CGF (n=17) | Çankaya et al. (2020), Tazegül et al. (2022), Mitra (2022), Bozkurt et al. (2022), Vaid et al. (2021), Shoukheba et al. (2021), Li et al. (2020), Huang et al. (2018), Talaat et al. (2018), Jia (2022), Lin HY (2016), Lin Y (2020), Ghasemirad et al. (2023), Lin (2021), Buffoli et al. (2021), Li (2022), Kavitha et al. (2022) |
